# Adjuvant Second-Dose Chemotherapy before Surgery for Patients with Locally Advanced Rectal Malignancy Is Not Beneficial: A Systematic Review and Meta-Analysis

**DOI:** 10.1155/2017/1373092

**Published:** 2017-08-01

**Authors:** Min Chen, Xue Song, Liang-zhou Chen, Lin Xu, Yi-pu Lu, Jin-song Zhang

**Affiliations:** Department of General Surgery, Xiamen Traditional Chinese Medicine (TCM) Hospital Affiliated to Fujian University of TCM, Xiamen 361009, China

## Abstract

**Background:**

Preoperative chemoradiotherapy is the standard treatment for patients with locally advanced rectal cancer, although tumor responses vary widely; some patients may achieve a pathologic complete response rate (pCR) after chemoradiotherapy. Controversy exists with regard to the efficacy of different preoperative combination chemotherapy regimens and neoadjuvant chemoradiotherapy, compared with chemoradiotherapy alone.

**Methods:**

PubMed, the Cochrane Library, and Embase databases were searched for comparative studies of patients with locally advanced rectal cancer that were published between January 1991 and January 2016. Efficacies of different preoperative combination chemotherapy regimens and neoadjuvant chemoradiotherapy (group A) were compared with chemoradiotherapy alone (group B) in a meta-analysis using Review Manager v5.2.

**Results:**

Three prospective randomized controlled trials and two prospective nonrandomized controlled trials comprising 444 cases were eligible for analysis. No significant difference was detected in the rate of pCR (50/223, 22.4% versus 35/223, 15.7%; relative risk, RR: 1.42 [95% confidence interval, CI: 0.97–2.09], *p* = 0.07) between the two groups. The rate of tumor regression was similar for both groups (122/203, 60.1% versus 111/203, 54.7%; RR: 1.11 [95% CI: 0.94–1.29], *p* = 0.22).

**Conclusions:**

Adjuvant chemotherapy with preoperative chemoradiotherapy did not significantly improve the rate of pCR nor the rate of T and N downstaging.

## 1. Background

Colorectal cancer is one of the most common cancers worldwide and a leading cause of cancer death [1, 2]. Previously, patients with colorectal cancer who underwent operative resection had a high rate of local recurrence. However, with the introduction of new operative procedures for total mesorectal excision (TME), the envelope of lymphovascular fatty tissue and the mesorectum [3] was completely excised, leading to a significant decrease in the local recurrence rate of colorectal cancer [4, 5]. Besides improvements in surgical technique, adjuvant therapy regimens provided excellent complementary therapy to reduce local recurrence rates of colorectal cancer.

Preoperative radiotherapy combined with TME can cut the 10-year local recurrence rate by more than half—relative to that with TME alone [6]. Locally advanced rectal cancer refers to clinical T3 N0 (or any-T, but N1–N2 disease), and approximately 70% of rectal cancers without metastasis are locally advanced [7], which emphasizes the necessity of neoadjuvant chemotherapy management of locally advanced rectal cancers. Although preoperative radiotherapy can significantly decrease local recurrence rates, it cannot effectively downstage the tumor itself, regardless of whether short-course [8–10] or conventionally fractionated radiotherapy with delayed TME is adopted as the treatment strategy [11]. As the combined effect of radiotherapy and chemotherapy has been noted experimentally [12], systemic chemotherapy was introduced in combination with preoperative radiotherapy to constitute neoadjuvant chemoradiotherapy.

Comparison of outcomes between patients receiving chemoradiotherapy and those receiving preoperative radiotherapy alone indicated that the local recurrence rate was significantly lower in the preoperative chemoradiotherapy group [13]. Moreover, the local recurrence rate of colorectal cancer was significantly decreased in the preoperative, as compared with the postoperative, chemoradiotherapy group [14]. Worldwide, chemoradiotherapy followed by TME has become the standard treatment for locally advanced rectal cancer. A number of recent studies have explored modified neoadjuvant chemoradiotherapy regimens, wherein a common strategy involves adjuvant chemotherapy either before or after chemoradiotherapy such that patients receive a sufficient therapeutic radiation dose as well as the intensity of chemotherapy. Despite being studied in several trials, conflicting results have been reported with these novel treatment regimens, and only some reports noted that additional chemotherapy increased the rate of pathologic complete response (pCR) [15, 16].

This meta-analysis of comparative trials was conducted to evaluate the effect of adjuvant chemotherapy in combination with chemoradiotherapy, as compared with chemoradiotherapy alone, on pCR in locally advanced rectal cancer.

## 2. Methods

### 2.1. Search Strategy

A comprehensive literature search was conducted in January 2016. Studies published between January 1991 and prior to January 2016 were identified in the following databases: Embase, PubMed, and the Cochrane Library, without restrictions of the region, publication type, or language. The following MeSH terms and their combinations were searched [Title/Abstract]: locally advanced, rectal/rectum, cancer/tumour/tumor/neoplasm, and chemoradiation/radiochemotherapy/chemoradiotherapy. Furthermore, the related “Articles” function in Review Manager was used to broaden the search. The computerized search was supplemented with a manual examination of reference lists of all retrieved studies, review articles, and conference abstracts. If multiple reports described the same populations, the most recent or complete report was used.

The full electronic search strategy used the following search terms for PubMed: (locally advanced) AND (rectal) OR rectum AND (cancer) OR (tumour) OR tumor) OR neoplasm OR “Rectal Neoplasms” [MeSH] AND (chemoradiation) OR radiochemotherapy) OR chemoradiotherapy) OR “Chemoradiotherapy” [MeSH].

### 2.2. Inclusion and Exclusion Criteria

All randomized controlled trials (RCTs) available as full-text or conference abstracts, prospective comparative studies that compared the efficacy of combined adjuvant chemotherapy and chemoradiotherapy with chemoradiotherapy alone in locally advanced rectal cancer, and studies with rate of pCR as one of the outcomes were included. Retrospective and noncomparative studies, editorials, letters to the editor, review articles, case reports, or preclinical studies were excluded.

### 2.3. Data Collection

The predefined information from the included studies was extracted and summarized independently by two investigators (JS Zhang and YP Lu). Information about the characteristics of the study population and relevant outcomes was recorded. Disagreements pertaining to data abstraction were resolved through discussion and, if unresolved, arbitrated by a third investigator (X Song) to reach consensus.

The incidence of pCR was selected as the primary outcome. pCR refers to the absence of tumor cells in the surgical specimen, including both at the primary tumor site and regional lymph nodes (ypT0N0). The secondary outcome was the downstaging effect, which was defined as the rate of posttreatment reduction of pathological stage (including both T and/or N categories) as compared with the pretreatment clinical stage.

Guidelines of the Cochrane Collaboration were used to assess the quality of the RCTs, [17] which were evaluated by six categories: sequence generation, allocation concealment, blinding, incomplete outcome data, selective outcome reporting, and other biases. Blinding was deemed to confer a low risk of bias if outcome assessors were blinded to the treatment information. The modified Newcastle-Ottawa scale was used to assess the quality of the prospective non-RCTs across three factors: patient selection, comparability of study groups, and assessment of outcome. A score of 0–9 allocated stars was used to evaluate the quality of nonrandomized controlled trials. RCTs and studies that received six or more stars were considered to be of high quality and were included in the meta-analysis.

### 2.4. Statistical Analysis

All statistical analyses were conducted using Review Manager v5.2 (Cochrane Collaboration, Oxford, England). A fixed model was used if there was no evidence of heterogeneity; otherwise, a random-effects model was used. Statistical heterogeneity between studies was assessed using the chi-squared test, with significance set at *p* < 0.10 and heterogeneity quantified using the *I*^2^ statistic. Relative risk (RR) was calculated for each trial from the number of evaluable patients, and all results were reported with 95% confidence intervals (CI). The *p* value for the overall effect was calculated using the *z*-test, with significance set at *p* < 0.05. A sensitivity analysis was conducted. Publication bias was not assessed due to the small number of trials.

## 3. Results

### 3.1. Trial Characteristics

**Figure 1:**
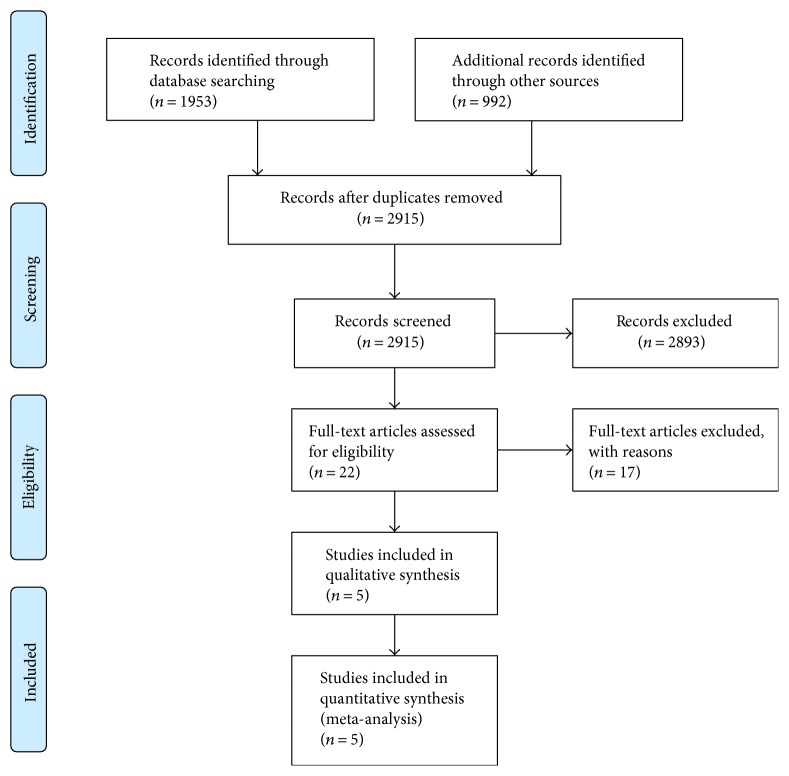
Flow diagram of trial selection process for inclusion in the meta-analysis.

The trial selection process is shown in Figure 1. Three RCTs and two nonrandomized trials that met the predefined inclusion criteria were eligible for final analysis. These studies included 223 patients who received preoperative adjuvant chemotherapy and chemoradiotherapy (group A) as well as 223 patients who received preoperative chemoradiotherapy alone (group B). The characteristics of these five studies are shown in Table 1. The risks of biases of three RCTs and the two nonrandomized studies are shown in Tables 2 and 3, respectively.

### 3.2. Results of Meta-Analysis

#### 3.2.1. Pathologic Complete Response

**Figure 2:**
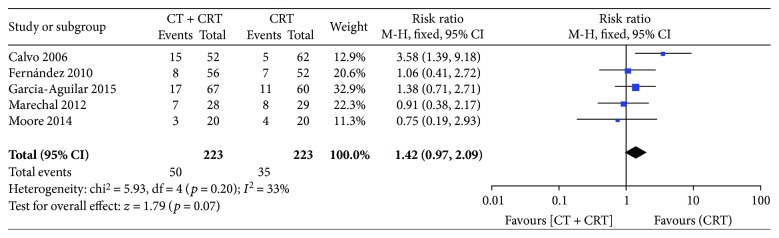
Forest plot for pCR. A Mantel-Haenszel fixed-effects model was used for meta-analysis. Risk ratios are shown with 95% confidence interval.

All of the included studies reported pCR rates that were 22.4% (50/223) and 15.7% (35/223) for group A and group B, respectively. No significant difference was detected in the rate of pCR (RR: 1.42 [95% CI: 0.97–2.09], *p* = 0.07) between the two groups (Figure 2).

#### 3.2.2. Downstaging

**Figure 3:**
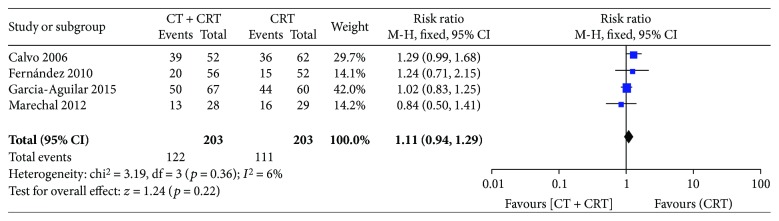
Forest plot for downstaging. A Mantel-Haenszel fixed-effects model was used for meta-analysis. Risk ratios are shown with 95% confidence interval.

Rates of downstaging were reported in four of the included studies and were 60.1% (122/203) in group A, but 54.7% (111/203) in group B. The addition of chemotherapy to chemoradiotherapy did not significantly improve the rate of downstaging (RR: 1.11 [95% CI: 0.94–1.29], *p* = 0.22; Figure 3).

### 3.3. Sensitivity Analysis and Publication Bias

Sensitivity analysis was conducted to determine the significance of each set of results. Three RCTs were included in the sensitivity analysis (Table 4), but there was no statistically significant change with regard to the outcome. Publication bias was not assessed due to the small number of trials.

## 4. Discussion

Chemoradiotherapy followed by TME in combination with postoperative adjuvant chemotherapy is a standard treatment for locally advanced rectal cancer. A limitation of this treatment is the need for patient compliance to postoperative adjuvant chemotherapy. Less than 50% of patients usually receive postoperative adjuvant chemotherapy [18]. A common therapeutic strategy is to add chemotherapy before or after chemoradiotherapy to improve compliance with chemotherapy to ensure patients receive a sufficient therapeutic dose and intensity of chemotherapy.

Patients with colorectal cancer respond to chemoradiotherapy differently; some patients may achieve a pCR after chemoradiotherapy, which suggests better prognostic outcomes. Maas et al. reported that the 5-year crude disease-free survival (DFS) was 83.3% in patients with pCR (61/419 patients had disease recurrence) and 65.6% in those without pCR (*p* < 0.0001) [19] Kuo et al. reported that the rate of recurrence was significantly different between the pCR and residual tumor groups (5.6% versus 31.1%; *p* = 0.002). The 5-year DFS was significantly better in the pCR group than in the residual tumor group (93% versus 66%; *p* = 0.0045) [20]. Chari et al. reported that, during the follow-up period, 39 of the 43 patients remained alive (median follow-up 25 months), and only 1 of the 11 patients with complete histologic response developed recurrent disease. Six of the 32 patients with residual disease (two with positive nodes) developed metastatic disease during follow-up (time to diagnosis: median 10 months, range 3–15 months) [21].

Approximately 15%–27% of patients with colorectal cancer can achieve a complete response; however, this percentage is not considered indicative of successful treatment, and increasing this proportion remains a major clinical challenge [19]. Therefore, we conducted a meta-analysis of comparative trials to evaluate the effect of adjuvant chemotherapy administered in combination with chemoradiotherapy on pCR in locally advanced rectal cancer.

The current review addressed the question whether adjuvant chemotherapy with preoperative chemoradiotherapy further improved the rate of pCR. We identified three RCTs and two nonrandomized trials that compared preoperative chemotherapy plus chemoradiotherapy against chemoradiotherapy alone in locally advanced rectal cancer. A meta-analysis of their results showed that additional chemotherapy did not significantly increase the rate of pCR, compared with chemoradiotherapy alone. Furthermore, adjuvant chemotherapy with preoperative chemoradiotherapy did not improve the rate of downstaging. However, another aspect was that this therapeutic modality did not increase the rate of grade 3-4 toxicity.

One study indicated that if preoperative radiotherapy is to be effective, then the biologically equivalent dose should be at least 20 Gy [22]. Furthermore, another study reported that different doses of preoperative radiotherapy (≥30 Gy) could have anticancer biological effects [23]. All five studies were reviewed with a biologically equivalent dose > 45 Gy, although the doses and fractionations of radiotherapy were different and radiotherapy regimens were identical in both groups in all of the studies. According to the results of our meta-analysis, we infer that radiotherapy doses cannot influence changes in the rate of pCR and T/N downstaging.

Boost radiation was undertaken in two studies and, despite the dose and regimen of the radiotherapy being different, results of these studies showed that adjuvant chemotherapy with chemoradiotherapy significantly improved the rate of pCR. However, no benefit in the rate of pCR was conferred with the addition of a boost radiotherapy dose [24, 25]. This difference may be attributed to the adjuvant chemotherapy administered in combination with the boost radiation. A meta-analysis of the results of these two studies using the Mantel-Haenszel random-effects model did not reveal a significant conclusion. Thus, the effect on the rate of pCR of such adjuvant chemotherapy and boost radiotherapy dose in combination with chemoradiotherapy remains uncertain. Indeed, it does not appear to be clinically sound.

A meta-analysis has shown that an interval longer than 6 to 8 weeks between chemoradiotherapy and surgery could significantly improve pCR [26]. The interval between chemoradiotherapy and surgery in four of the five studies was consistent, except for a study by Garcia-Aguilar (11.1 weeks versus 8.5 weeks). However, in this study, the interval in both groups was longer than 6 to 8 weeks, and the difference in intervals did not affect the overall results of the meta-analysis.

Three trials used induction chemotherapy before chemoradiotherapy. Induction chemotherapy involves the application of chemotherapy with sufficient dose and intensity. Chau and colleagues reported that induction with capecitabine and oxaliplatin before chemoradiotherapy and TME may increase the tumor response rate up to 97%. Moreover, after induction with capecitabine/oxaliplatin, the radiologic response rate was 88% [27].

We included these three studies in the meta-analysis and found that induction chemotherapy before chemoradiotherapy did not significantly improve the overall pCR. Two trials used chemotherapy after chemoradiotherapy. The rationale of this strategy is that the tumor response to chemoradiotherapy is time-dependent, whereas shorter intervals may interrupt ongoing tumor necrosis [28]. Moreover, using chemotherapy after chemoradiotherapy allowed the administration of sufficient dose and the intensity of chemotherapy and, thus, prolonged the interval between chemoradiotherapy and surgery; this allowed enough time for a tumor response to achieve pCR. The same conclusion can be drawn from these two studies: adjuvant chemotherapy after chemoradiotherapy did not improve the pCR significantly.

Three studies used chemotherapy (which contained oxaliplatin) before chemoradiotherapy. Combination therapy with oxaliplatin could enhance the antitumor effect of 5-fluorouracil compared with 5-fluorouracil monotherapy; moreover, it could function as a potent radiosensitizing agent [29]. Finally, these three studies showed that the addition of oxaliplatin-containing chemotherapy to chemoradiotherapy did not improve the pCR significantly.

The main limitation of our review is that only five published manuscripts form the basis of its meta-analysis; moreover, two of the five studies were not RCTs. This may have potentially increased the risk of bias due to inadequate randomization. One of the RCTs included was only available as a conference abstract, which does not provide complete data, and was, therefore, not considered a scientific evidenced-based study. Furthermore, the regimen of adjuvant chemotherapy and chemoradiotherapy as well as the interval between chemoradiotherapy and surgery differed across the five studies, which may have resulted in an underestimation of the overall therapeutic efficacy. Finally, the number of patients evaluated is small (only 223 patients in each arm), which may be inadequate to identify distinct differences in the rate of pCR. Therefore, another meta-analysis that includes other studies with more data will be required in the future to elucidate further on the differences between the studied chemotherapeutic and chemoradiotherapeutic regimens.

## 5. Conclusions

The addition of a second dose of chemotherapy to the primary chemoradiotherapy regimen before TME in locally advanced rectal cancer did not change the rate of pCR or that of T and N downstaging. Given the inherent limitations of this meta-analysis, additional large-scale RCTs that evaluate the outcomes and safety with adjuvant chemotherapy and chemoradiotherapy are warranted.

## Figures and Tables

**Table 1:** Characteristics of studies included in the meta-analysis.

Study	Year	Country	Additional CT regimen	CT regimen in CRT	CT before or after CRT	Boost RT	Total radiation (Gy)	Surgery	Group	Interval^#^ (weeks)	*N* (M/F)	Age (years)	cT stage			cT_X_N+
													T2	T3	T4	
Fernández-Martos et al. [16]	2010	Spain	CAPOX 4 cycles	CAPOX	Before	No	50.4	TME	CT + CRT	5-6	56 (39/17)	60 (38–76)		18	7	31
									CRT	5-6	52 (34/18)	62 (42–75)		17	3	31
Marechal et al. [30]	2012	Belgium	FOLFOX6 (1) 2 cycles	5-FU	Before	No	45	TME	CT + CRT	6–8	28 (21/7)	62 (22–80)	1	25	2	26
									CRT	6–8	29 (16/13)	62 (44–79)	3	23	3	25
Moore et al. [31]	2014	Australia	5FU + leucovorin 3 cycles	5-FU	After	NR	50.4	NR	CT + CRT	10	20	NA	NA	NA	NA	NA
									CRT	10	20	NA	NA	NA	NA	NA
Calvo et al. [32]	2006	Spain	FOLFOX4 2 cycles	Tegafur	Before	Yes	≥50.4	Safety distal margin distance or TME	CT + CRT	4–6	52 (38/14)	69 (37–80)	4	43	5	24
									CRT	4–6	62 (43/19)	66 (26–82)	2	53	7	27
Garcia-Aguilar et al. [33]	2015	USA	FOLFOX6 (2) 2 cycles	5-FU	After	Yes	≥50.4	TME	CT + CRT	11.1	67 (37/30)	56 (32–84)	6	60	1	55
									CRT	8.5	60 (37/23)	57 (34–87)	2	57	1	40

Note: CAPOX, capecitabine 2000 mg/m^2^ 14/21 days and oxaliplatin 130 mg/m^2^ 1/21 days; FOLFOX6 (1), oxaliplatin 100 mg/m^2^ 1/14 days, folinic acid 400 mg/m^2^ 1/14 days, 5-FU 400 mg/m^2^ 1/14 days, and 5-FU 2000 mg/m^2^ CVI over 46 h/14 days. FOLFOX4, oxaliplatin 85 mg/m^2^ 1/14 days, folinic acid 200 mg/m^2^ 1-2/14 days, 5-FU 400 mg/m^2^ 1/14 days, and 5-FU 600 mg/m^2^ CVI in 22 h/14 days. FOLFOX6 (2), oxaliplatin 85 mg/m^2^ 1/14 days, folinic acid 400 or 200 mg/m^2^ 1/14 days, 5-FU 400 mg/m^2^ 1/14 days, and 5-FU 2400 mg/m^2^ CVI over 46 h/14 days. ^#^Interval between surgery and CRT. CT, chemotherapy; CRT, chemoradiotherapy; RT, radiotherapy; TME, total mesorectal excision; *N*, patient number; M, male; F, female; NA, not available.

**Table 2:** Risk of bias in the prospective randomized controlled studies.

Study	Adequate random sequence generation	Allocation concealment	Blinding of participants and personnel	Incomplete outcome data	Selective outcome reporting	Other biases
Fernández-Martos et al. [16]	Yes	No	Yes	Yes	Yes	Yes
Marechal et al. [30]	Yes	No	Yes	Yes	Yes	Yes
Moore et al. [31]	No	No	Yes	Yes	Yes	Yes

**Table 3:** Risk of bias in the nonrandomized studies using modified Newcastle-Ottawa scale.

Study	Selection			Comparability		Outcome		Quality score
	Assign for treatment^△^	Representative treatment group	Representative reference group	Comparable for 1, 2, 3, 4, and 5^∗^	Comparable for 6, 7, 8, and 9^∗^	Assessment of outcome	Adequate follow-up	
Calvo et al. [32]	No	Yes	Yes	1, 2, and 3	6, 7, and 8	Yes	Yes	★★★★★★
Garcia-Aguilar et al. [33]	No	Yes	Yes	1, 2, 3, and 4	6, 7, and 8	Yes	Yes	★★★★★★

Comparability variables: 1, age; 2, gender; 3, TN; 4, total mesorectal excision; 5, radial margin status; 6, distance from anal verge; 7, radiotherapy technique; 8, total dose of radiotherapy; and 9, adjuvant chemotherapy. ^∗^If all characteristics were comparable, two stars; if >2 characteristics were comparable, one star; if <2 characteristics were comparable, no star. ^△^The article provided the details of criteria for adequate random assignment.

**Table 4:** Comparison of sensitivity analysis of pCR in the three RCTs.

Outcomes of interest	Number of studies	CT + CRT number	CRT number	RR (95% CI)	*p* value	Study heterogeneity		
						df	*I* ^2^ (%)	*p* value
pCR	3	104	101	0.93 (0.52–1.66)	0.81	2	0	0.92
